# Hours and Miles: Patient and Health System Implications of Transfer for Psychiatric Bed Capacity

**DOI:** 10.5811/westjem.2016.9.30443

**Published:** 2016-10-07

**Authors:** Amy M. O’Neil, Annie T. Sadosty, Kalyan S. Pasupathy, Christopher Russi, Christine M. Lohse, Ronna L. Campbell

**Affiliations:** * Mayo Clinic, Department of Emergency Medicine, Rochester, Minnesota; † Mayo Clinic, Division of Health Care Policy and Research and Robert D. and Patricia E. Kern Center for the Science of Health Care Delivery, Rochester, Minnesota; ‡ Mayo Clinic, Division of Biomedical Statistics and Informatics, Rochester, Minnesota

## Abstract

**Introduction:**

An increasing number of behavioral health (BH) patients are presenting to the emergency department (ED) while BH resources continue to decline. This situation-may lead to more external transfers to find care.

**Methods:**

This is a retrospective cohort study of consecutive patients presenting to a tertiary care academic ED from February 1, 2013, through January 31, 2014. Patients were identified through electronic health record documentation of psychiatric consultation during ED evaluation. We reviewed electronic health records for demographic characteristics, diagnoses, payer source, ED length of stay, ED disposition, arrival method, and distance traveled to an external facility for inpatient admission. Univariable and multivariable associations with transfer to an external facility in comparison with patients admitted internally were evaluated with logistic regression models and summarized with odds ratios (OR).

**Results:**

We identified 2,585 BH visits, of which 1,083 (41.9%) resulted in discharge. A total of 1,502 patient visits required inpatient psychiatric admission, and of these cases, 177 patients (11.8%; 95% CI = [10.2–13.5]) required transfer to an external facility. The median ED length of stay for transferred patients was 13.9 hours (interquartile range [IQR], 9.3–20.2 hours; range, 3.0–243.0 hours). The median distance for transport was 83 miles (IQR, 42–111 miles; range, 42–237 miles). In multivariable analysis, patients with suicidal or homicidal ideation had increased risk of transfer (odds ratio [OR] [95% CI], 1.93 [1.22–3.06]; *P*=0.005). Children younger than 18 years (OR [95% CI], 2.34 [1.60–3.40]; *P<*0.001) and adults older than 65 years (OR [95% CI], 3.46 [1.93–6.19]; *P*<0.001) were more likely to require transfer and travel farther to access care.

**Conclusion:**

Patients requiring external transfer for inpatient psychiatric care were found to have prolonged ED lengths of stay. Patients with suicidal and homicidal ideation as well as children and adults older than 65 years are more likely to require transfer.

## INTRODUCTION

The population of patients in need of mental health care continues to grow despite increasing limitations in resources available for these patients.^1^–^5^ Behavioral health (BH) patients presenting to the emergency department (ED) who require transfer to other facilities, children and adults older than 65 years, and those with a history of violence or cognitive disorder are more likely to have prolonged ED length of stay (LOS) while a facility with capacity to care for them is identified.^6^–^8^ Long LOS strains ED resources and places the patient at increased risk of harm by prolonging the stay in a facility that may be inadequately equipped to prevent patient self-harm and other adverse events, including placing staff safety at risk.^9^–^11^

The necessity for patient transfer to an external psychiatric facility results in prolonged ED LOS.^3^,^7^ Although prolonged lengths of stay for BH patients have been well documented,^3^,^6^–^8^ little is known about the subsequent effects on the patients themselves. Specifically, the distance that patients must be transported to be admitted has not been studied. The objectives of this study were to characterize the ED patients who require external transport to psychiatric facilities and to track the distances they must travel due to insufficient local psychiatric inpatient capacity.

## METHODS

### Study Design and Setting

This was a retrospective cohort study of consecutive patients who presented to the ED of the Mayo Clinic Hospital - Rochester, Saint Marys Campus from February 1, 2013, through January 31, 2014. The hospital has a tertiary care academic ED with 73,000 annual patient visits and includes a dedicated psychiatric hospital consisting of 73 psychiatric beds divided among a child and adolescent unit (18 beds), an acute adult unit (25 beds), an adult mood disorders unit (16 beds), and a medical psychiatry and geriatric psychiatry unit (14 beds). The city of Rochester, Minnesota, also has the Community Behavioral Health Hospital, which has 16 beds for adult patients.

The Mayo Clinic Institutional Review Board reviewed and accepted the study protocol before study initiation. The reporting of study results follows the reporting guidelines of Strengthening the Reporting of Observational Studies in Epidemiology (STROBE).^12^

### Selection of Participants

We identified all ED BH patients through documentation of a psychiatric consult during ED evaluation in their electronic health record. They were eligible for inclusion when they provided research authorization in accordance with Minnesota law.

### Data Collection and Outcome Measures

The electronic health record was reviewed for data that were retrospectively collected, including patient demographic characteristics (e.g., age), diagnosis, payer source, ED LOS, ED disposition, arrival method, and distance traveled to an external facility for inpatient disposition. All patient data could be electronically extracted using the electronic health record in use (PulseCheck version 5.4; Optum) and did not require manual chart review. We characterized patients as *transferred* if they were discharged to a nonlocal external facility. Those who required admission and were hospitalized at the affiliated hospital were characterized as *admitted*. We subsequently calculated the distance to transfer sites on the basis of the number of miles reported on Google Maps.

### Statistical Analysis

We summarized continuous features with median, interquartile range (IQR), and absolute range; categorical features were summarized with frequency count and percentage. Comparisons of patients who received a psychiatric consultation and were discharged from the ED with patients who received a psychiatric consultation and were admitted for psychiatric care were evaluated with Wilcoxon rank sum and χ^2^ tests. We evaluated the differences in distance to the external facility between age groups with Wilcoxon rank sum tests. Univariable and multivariable associations with transfer to an external facility were evaluated with logistic regression models and summarized with odds ratios (ORs) and 95% confidence intervals (CIs). We conducted multivariable associations to determine if the significant associations observed on univariable analysis remained after multivariable adjustment. All variables of interest were used in both univariable and multivariable analyses. Univariable associations with transfer to an external facility were subsequently evaluated for the adult and pediatric cohorts separately. We performed statistical analyses with version 9.3 of the SAS software package (SAS Institute Inc). All tests were two-sided, and *P* values less than 0.05 were considered statistically significant.

## RESULTS

During the study period, we identified 2,585 ED patient visits involving an ED consultation by psychiatry services. Multiple patients presented to the ED on more than one occasion. Of the 2,585 ED visits, there were 1,981 distinct patients seen in the ED. Of the ED visits, 1,083 (41.9%) were patients evaluated and discharged from the ED and 1,502 (58.1%) were patients evaluated and determined to require inpatient psychiatric care. In the second group, 1,325 patients (83.9%) were admitted to the affiliated hospital, 65 (4.3%) were transferred to the local community behavioral health hospital, and 177 patients (11.8%; 95% CI = [10.2–13.5]) required transfer to a nonlocal external facility (Table 1).

The characteristics of admitted patients were similar to the dismissed cohort (Table 1). The median age of BH patients was in the early third decade. More than one-half (54.0%) of the patients were female. Most patients arrived to the ED by personal transport. The cohorts differed in payer source, as well as diagnosis. Admitted patients also had a significantly longer LOS than the non-admitted patients.

The median distance required for transfer to outside facilities was 83 miles (IQR, 42–111; range, 41–280 miles) (Figure). Fifty patient transports (28.2%; 95% CI = [21.9–35.6]) were within 50 miles, 63 (35.6%; 95% CI = [28.9–43.2]) were transferred between 50 and 100 miles, and 46 (26.0%; 95% CI = [19.8–33.2]) were transferred between 100 and 200 miles. Children and patients older than 65 years also required longer transport distances. The median distance to the external facility for patients younger than 18 years (102 [IQR, 83–141; range, 72–262] miles) was significantly greater than for patients aged 18 to 65 years (60 [IQR, 42–85; range, 41–280] miles; *P*<0.001). The distance for patients older than 65 years (83 [IQR, 59.5–144.5; range, 42–226] miles) also was significantly greater than for patients aged 18 to 65 years (*P*=0.04).

The median ED LOS for patients transferred to an external psychiatric facility was 13.9 hours (IQR, 9.3–20.2; range, 3.0–243.0 hours). Patients who did not require transfer and were admitted to inpatient psychiatric services in house had significantly shorter stays (4.4 [IQR, 3.4–6.7; range, 0.3–76.0] hours; *P*<0.001).

The characteristics of patients who were admitted to our hospital vs those who required transfer to an external facility for admission are summarized in Table 2, with the results of the univariable and multivariable models to predict transfer to an external facility. The multivariable analysis indicated that patients with suicidal or homicidal ideation had increased risk of requiring transport to an external facility (OR [95% CI], 1.93 [1.22–3.06]; *P*=0.005). Patient age was also significantly associated with increased risk of patient transfer. Children younger than 18 years were more likely to require transfer than patients aged 18 to 65 years (OR [95% CI], 2.34 [1.60–3.40]; *P*<0.001). In addition, adults older than 65 years were more likely to require transfer to an external facility (OR [95% CI], 3.46 [1.93–6.19]; *P*<0.001). Lastly, patients with noncommercial medical insurance were more likely to be transferred to an external facility, independent of patient age (Medicare or Medicaid, OR [95% CI], 1.54 [1.04–2.27], *P*=0.03; self-pay/other, OR [95% CI], 2.08 [1.30–3.32], *P*=0.002).

We also analyzed associations with transfer to an external facility vs admission to our hospital in the subsets of adult and pediatric cohorts. In these univariable models, adults with a diagnosis of suicidal or homicidal ideation were still found to be more likely to be transferred to an external facility (OR [95% CI], 2.17 [1.23–3.81]; *P*=0.007); however, there was no longer a significant association in the pediatric population (OR [95% CI], 1.18 [0.56–2.51]; *P*=0.67) (Table 3).

## DISCUSSION

Nearly 12% of BH patients required transport to an external psychiatric facility. Other investigators evaluating ED LOS for psychiatric patients have reported significantly higher rates of external transfer (37%–46%) for patients presenting with a mental health concern.^7^,^13^ Similar to other studies, our analysis found that BH patients requiring transport to an external psychiatric facility have prolonged LOS compared with those discharged or admitted locally.^14^,^15^ In our study, this difference was approximately three times longer (4.4 vs 13.9 hours). Chang et al^14^ demonstrated a median LOS approximately 2.5 times longer (2.5 vs 6.3 hours). Although most of the protracted LOS instances were measured in hours, some were measured in days (longest period, >10 days).

Our study reinforces some of the data previously published on factors affecting ED LOS. Nevertheless, this is the first study, to our knowledge, to characterize the patient experience—including distances that ED patients are transported to access inpatient psychiatric care—when local care is unavailable.

We found that certain patients had a greater predisposition for external transfer for inpatient psychiatric hospitalization than other patients, and transportation distances were considerable for patients requiring this transfer. Adults older than 65 years, children, patients with suicidal or homicidal ideation, and patients with noncommercial medical insurance were more likely to require transport to an external facility. In addition, when external transport was required, the older adults and the children were transported farther distances to access inpatient psychiatric care. Although the median travel distance was 83 miles, 10% of transports spanned more than 200 miles. This may be due in part to the location of our facility, which has largely rural surrounding communities. The closest location from our facility for inpatient psychiatric care is 41 miles away. This problem, however, is not unique to our institution. Between 1990 and 2008, the number of hospital or residential mental health organizations decreased by 812, with a loss of 86,515 beds.^15^ As closures of psychiatric facilities throughout the country continue, many hospitals likely face similar, if not longer, distances to the next inpatient psychiatric facility. These distant hospitalizations can place substantial burdens on patients and their family members.

Patient age was strongly associated with increased risk of need for external transfer. Children were more likely to require transfer. This need may be due to the overall lack of pediatric inpatient psychiatric beds available in the region. Minnesota has approximately seven adult inpatient psychiatric beds for every pediatric bed.^16^ National data indicate that adults and adolescents have a similar prevalence of mental illness, and in a recent report, adolescents had a higher rate of serious mental illness than adults (8.0% vs 5.8%).^17^ Children requiring psychiatric admission have the added stress of prolonged ambulance transport to an unknown facility and may have to travel without parental supervision. Parents are faced with the challenge of arranging their own transportation to visit their child and coordinating leave from their employer and care for other dependents. Although adult psychiatric care facilities have declined over the years, pediatric treatment centers have not experienced a similar trend and in fact have increased in number nationally—so, too, have the number of specialists certified in child and adolescent mental health care.^18^ While this is a positive trend, resources will need to continue to grow in order to meet the growing needs of the population.

Adults older than 65 years are also more likely to require transfer to an external facility than younger adults. In our institution, a limited number of geriatric psychiatric beds are available. In addition, a limited number of medical psychiatric beds are available to care for the higher rate of comorbid medical conditions in this population. Estimates report that more than 20% of geriatric patients have mental disorders, and as the U.S. population continues to age, this number is expected to double over the next 30 years.^19^ Cromwell and Maier^19^ demonstrated that these medical psychiatric units and geriatric psychiatric units require the most staff hours per patient per day compared with general adult units, psychiatric intensive care units, and dual psychiatric and substance-abuse units.^20^ The burgeoning geriatric population and the increased requisite psychiatric resources likely will pose challenges for inpatient placement and may continue to increase the transfer rate for these patients.

Adult patients who received a diagnosis including suicidal or homicidal ideation were almost twice as likely to be transferred to another facility. This finding was not seen in the pediatric population. This may be due to the increased resources such as video-monitored rooms and additional staffing needed to care for potentially violent adult patients.

Finally, an association was found between transfer to an external facility and a noncommercial payer source. The reasons for this finding are not clear, because the payer source is not considered in the process of identifying either outside facilities for transfer or patients for admission to our psychiatric hospital. Thus, lack of commercial insurance may correlate with other factors not accounted for in our model. Further studies are needed to more clearly understand this association.

This study is limited by being isolated to a single tertiary care setting with a relatively large internal psychiatric inpatient capacity. Hospitals in a larger urban setting may experience different trends in the association between increased LOS and patient transfers. In addition, this study is limited in that data were extracted electronically rather than by manual individual chart review. Hence, the study is limited by what data could be extracted electronically from the health record.

## CONCLUSION

Inadequate local and regional psychiatric hospital capacity results in significantly prolonged ED LOS and puts many patients at risk for transfer outside their local community for care. Patients with suicidal and homicidal ideation, patients older than 65 years, and children are at significantly increased risk for requiring transfer to an external facility for inpatient psychiatric care. Delays in transfers to distant facilities for inpatient psychiatric care strain the ED system, and the transfers place additional stress on patients and their families. A thorough evaluation of the BH system is needed to better address patient needs for inpatient psychiatric care.

## Figures and Tables

**Figure:**
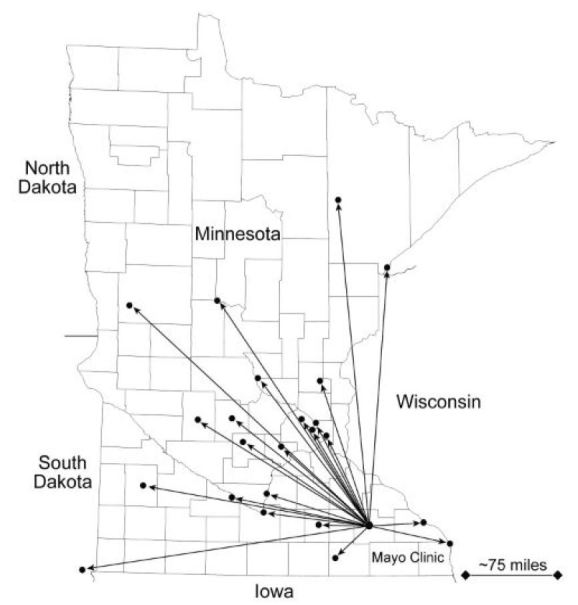
Locations for patients requiring transfer to an external facility for inpatient psychiatric care. Median transport distance was 83 miles; the longest distance was 280 miles.

**Table 1:** Summary of patient characteristics collected for behavioral health visits to the emergency department.

Characteristic^a^	All ED BH patient visits (n=2,585)	Patient visits resulting in discharge (n=1,083)	Patient visits resulting in psychiatric admission (n=1,502)	*P* value
Age, median (IQR; range), y	31 (20–47; 4–93)	30 (20–46; 4–93)	32 (20–48; 5–90)	0.07
Age, y				
<18	510 (20)	212 (20)	298 (20)	0.96
18–65	1,941 (75)	816 (75)	1,125 (75)	
>65	134 (5)	55 (5)	79 (5)	
Gender				
Female	1,392 (54)	585 (54)	807 (54)	0.88
Male	1,193 (46)	498 (46)	695 (46)	
Mode of arrival	(n=2,579)^b^		(n=1,497)^b^	
Personal transport	1,761 (68)	728 (67)	1,033 (69)	0.51
EMS	494 (19)	209 (19)	285 (19)	
Law enforcement	324 (13)	145 (13)	179 (12)	
Payment type				
Commercial	945 (37)	357 (33)	588 (39)	<0.001
Medicare or Medicaid	1,132 (44)	461 (43)	671 (45)	
Other/self-pay	508 (20)	265 (24)	243 (16)	
Diagnosis				
Mood disorder	629 (24)	246 (23)	383 (26)	<0.001
Suicidal or homicidal ideation	848 (33)	219 (20)	629 (42)	
Altered thought processes	243 (9)	93 (9)	150 (10)	
All others	865 (33)	525 (48)	340 (23)	
Transfer to external facility	177 (7)	0	177 (12)	
LOS, median, h	4.4	3.8	4.8	<0.001
LOS, range (IQR), h	0.2–243.0 (3.1–7.2)	0.2–74.6 (2.7–5.8)	0.3–243.0 (3.5–8.9)	

*BH*, behavioral health; *ED*, emergency department; *EMS*, emergency medical services; *IQR*, interquartile range; *LOS*, length of stay.

a Values are presented as number (percentage) of patients unless specified otherwise.

b Sample size for characteristics with missing data.

**Table 2:** Univariable and multivariable associations of behavioral-health patient characteristics with transfer to external facility.

			Univariable		Multivariable	
				
Characteristic^a^	No transfer (n=1,325)	Transfer (n=177)	OR (95% CI)	*P* value	OR (95% CI)	*P* value
Age, y						
<18	244 (18)	54 (31)	2.20 (1.54–3.14)	<0.001	2.34 (1.60–3.40)	<0.001
18–65	1,022 (77)	103 (58)	1.0 (reference)		1.0 (reference)	
>65	59 (5)	20 (11)	3.36 (1.95–5.81)	<0.001	3.46 (1.93–6.19)	<0.001
Gender						
Female	709 (54)	98 (55)	1.0 (reference)		1.0 (reference)	
Male	616 (46)	79 (45)	0.93 (0.68–1.27)	0.64	0.97 (0.70–1.34)	0.84
Mode of arrival						
Personal transport	907 (69)	126 (71)	1.0 (reference)		1.0 (reference)	
EMS	252 (19)	33 (19)	0.94 (0.63–1.42)	0.78	0.94 (0.61–1.44)	0.77
Law enforcement	161 (12)	18 (10)	0.81 (0.48–1.36)	0.41	0.78 (0.45–1.33)	0.35
Payment type						
Commercial	534 (40)	54 (31)	1.0 (reference)		1.0 (reference)	
Medicare or Medicaid	584 (44)	87 (49)	1.47 (1.03–2.11)	0.04	1.54 (1.04–2.27)	0.03
Other/self-pay	207 (16)	36 (20)	1.72 (1.10–2.70)	0.02	2.08 (1.30–3.32)	0.002
Diagnosis						
Mood disorder	340 (26)	43 (24)	1.41 (0.86–2.32)	0.18	1.57 (0.93–2.63)	0.09
Suicidal/homicidal ideation	540 (41)	89 (50)	1.84 (1.18–2.87)	0.008	1.93 (1.22–3.06)	0.005
Altered thought processes	133 (10)	17 (10)	1.42 (0.75–2.69)	0.28	1.44 (0.75–2.77)	0.27
All others	312 (24)	28 (16)	1.0 (reference)		1.0 (reference)	

*EMS,* emergency medical services; *OR*, odds ratio.

a Values are presented as number (percentage) of patients unless specified otherwise.

**Table 3:** Univariable associations of characteristics with transfer to external facility for adult and pediatric patients.

	Adult patients (n=1,204)				Pediatric patients (n=298)			
		
Characteristic^a^	No transfer (n=1,081)	Transfer (n=123)	OR (95% CI)	*P* value	No transfer (n=244)	Transfer (n=54)	OR (95% CI)	*P* value
Age, y					NA	NA	NA	
18–65	1,022 (95)	103 (84)	1.0 (reference)					
>65	59 (5)	20 (16)	3.36 (1.95–5.81)	<0.001				
Gender								
Female	564 (52)	67 (54)	1.0 (reference)		145 (59)	31 (57)	1.0 (reference)	
Male	517 (48)	56 (46)	0.91 (0.63–1.33)	0.63	99 (41)	23 (43)	1.09 (0.60–1.97)	0.78
Mode of arrival^b^								
Personal transport	726 (67)	82 (67)	1.0 (reference)		181 (74)	44 (81)	1.0 (reference)	
EMS	225 (21)	28 (23)	1.10 (0.70–1.74)	0.68	27 (11)	5 (9)	0.76 (0.28–2.09)	0.60
Law enforcement	126 (12)	13 (11)	0.91 (0.49–1.69)	0.77	35 (14)	5 (9)	0.59 (0.22–1.59)	0.29
Payment type								
Commercial	392 (36)	26 (21)	1.0 (reference)		142 (58)	28 (52)	1.0 (reference)	
Medicare or Medicaid	510 (47)	69 (56)	2.04 (1.28–3.26)	0.003	74 (30)	18 (33)	1.23 (0.64–2.38)	0.53
Other/self-pay	179 (17)	28 (23)	2.36 (1.34–4.14)	0.003	28 (11)	8 (15)	1.45 (0.60–3.51)	0.41
Diagnosis								
Mood disorder	283 (26)	32 (26)	1.75 (0.95–3.23)	0.07	57 (23)	11 (20)	0.95 (0.38–2.37)	0.91
Suicidal or homicidal ideation	407 (38)	57 (46)	2.17 (1.23–3.81)	0.007	133 (55)	32 (59)	1.18 (0.56–2.51)	0.67
Altered thought processes^c^	128 (12)	17 (14)	2.06 (1.02–4.16)	0.045	54 (22)	11 (20)	1.0 (reference)	
All others	263 (24)	17 (14)	1.0 (reference)					
Length of stay, median (IQR; range), h^d^	4.4 (3.4–6.4; 0.3–76.0)	12.8 (8.6–18.2; 3.3–140.1)	1.47 (1.34–1.61)	<0.001	4.6 (3.2–15.7; 2.0–75.1)	15.9 (11.0–25.9;3.0–243.0)	1.18 (1.09–1.27)	<0.001

*EMS,* emergency medical services; *IQR,* interquartile range; *NA,* not applicable; *OR*, odds ratio.

a Values are presented as number (percentage) of patients unless specified otherwise.

b For adult patients, n=1,200; for pediatric patients, n=297.

c All other diagnoses were combined with this category for the analysis of pediatric patients.

d OR represents a 4-h increase in length of stay.
